# Determination of fluorine in herbs and water samples by molecular absorption spectrometry after preconcentration on nano-TiO_2_ using ultrasound-assisted dispersive micro solid phase extraction

**DOI:** 10.1007/s00216-017-0589-y

**Published:** 2017-08-29

**Authors:** Magdalena Krawczyk-Coda, Ewa Stanisz

**Affiliations:** Faculty of Chemical Technology, Poznan University of Technology, Berdychowo 4, 60-965 Poznań, Poland

**Keywords:** TiO_2_, Dispersive micro solid phase extraction, Ultrasound, Fluorine, HR-CS ET MAS

## Abstract

This work presents ultrasound-assisted dispersive micro solid phase extraction (USA DMSPE) for preconcentration of fluorine (F) in water and herb samples. TiO_2_ nanoparticles (NPs) were used as an adsorbent. The determination with slurry sampling was performed via molecular absorption of calcium monofluoride (CaF) at 606.440 nm using a high-resolution continuum source electrothermal absorption spectrometry (HR-CS ET MAS). Several factors influencing the efficiency of the preconcentration technique, such as the amount of TiO_2_, pH of sample solution, ultrasonication and centrifugation time and TiO_2_ slurry solution preparation before injection to HR-CS ET MAS, were investigated in detail. The conditions of detection step (wavelength, calcium amount, pyrolysis and molecule-forming temperatures) were also studied. After extraction, adsorbent with the analyte was mixed with 200 μL of H_2_O to prepare a slurry solution. The concentration limit of detection was 0.13 ng mL^−1^. The achieved preconcentration factor was 7. The relative standard deviations (RSDs, %) for F in real samples were 3–15%. The accuracy of this method was evaluated by analyses of certified reference materials after spiking: INCT-MPH-2 (Mixed Polish Herbs), INCT-SBF-4 (Soya Bean Flour), ERM-CAO11b (Hard Drinking Water) and TMDA-54.5 (Lake Ontario Water). The measured F contents in reference materials were in satisfactory agreement with the added amounts, and the recoveries were found to be 97–109%. Under the developed extraction conditions, the proposed method has been successfully applied for the determination of F in real water samples (lake, sea, tap water) and herbs.

## Introduction

Fluorine (F) in the environment, mainly as fluorides, represent about 0.06–0.09% of the earth’s crust [1]. Drinking water and food are defined as the primary sources of fluoride intake, but daily exposure depends heavily on the geographical area [2, 3]. In drinking water, it is considered as a beneficial component at levels of about 0.7 mg L^−1^. However, fluoride may be harmful if it exceeds 1.5 mg L^−1^. It is the limit set by the World Health Organization and is followed in many countries [1, 3, 4]. The most accessible form of fluoride and, hence, the most toxic is intake through drinking water [2].

In low concentrations, fluoride strengthens the tooth enamel [4]. Concentrations in the range of 1.5–4 mg L^−1^ result in dental fluorosis but higher values (4–10 mg L^−1^) may cause dental fluorosis to progress to skeletal fluorosis. Fluoride may displace hydroxide ions from hydroxyapatite, Ca_5_(PO_4_)_3_OH, the principal mineral constituent of teeth and bones, to form the harder and tougher fluorapatite, Ca_5_(PO_4_)_3_F. It makes the teeth and bones harder and more brittle [4]. It is also suggested that fluoride may combine with calcium forming fluorapatite during mineralisation resulting in a gradual reduction in the calcium content [5].

The determination of fluoride in water samples is usually performed using chromatographic methods, such as ion chromatography (IC) [6], gas chromatography (GC) [7] and high-performance liquid chromatography (HPLC) [8, 9] or electrochemical methods, such as ion selective electrodes (ISE) [10, 11], polarography [12] and voltammetry [13]. Spectroscopic methods, for example inductively coupled plasma-optical emission spectrometry (ICP-OES) or classical atomic absorption spectrometry (AAS), are not used for determination of F because of the very high ionisation potential (17.42 eV) and the resonance lines of the fluorine being below 100 nm. An alternative method for F determination is molecular absorption spectrometry (MAS) conduct with the use of high-resolution continuum source atomic absorption spectrometry (HR-CS AAS) [14–16]. This method is based on the formation of stable monofluorides of aluminium [17–19], gallium [20–25], calcium [26–28] or strontium [29]. Diatomic molecules can absorb defined energy from a continually emitting spectral radiation source, creating molecular absorption spectra. The molecular absorption spectra correspond to the molecular transitions between the different molecule states. The molecular absorption spectrum has more lines than atomic absorption spectrum. The line width of various molecular absorption lines is roughly equal to the atomic absorption lines and can be resolved and used for analysis by HR-CS AAS.

In our previous studies, SPE in micro dispersive mode was presented using ZnO [30] as well as Ag [31] and TiO_2_ [32] nanoparticles for the isolation and preconcentration of Ge, Hg and Hg species, respectively. In this paper, the combination of ultrasound-assisted DMSPE (USA DMSPE) with nano-TiO_2_ as an adsorbent and high-resolution molecular absorption spectrometry with electrothermal vaporisation of the calcium monofluoride (HR-CS ET MAS) is presented for F determination.

To the best of our knowledge, a procedure combining these approaches has not been reported yet. Direct addition of TiO_2_ as an adsorbent to the sample solution provided rapid adsorption onto the material under short ultrasonication time. After extraction, the material was separated from the sample solution and injected as a slurry solution to HR-CS ET MAS. The accuracy of the method was demonstrated by analysis of spiked certified reference materials. Finally, the developed procedure was applied for extraction and determination of F in water samples and herb samples.

## Materials and methods

### Instrumentation

A ContrAA 700 HR-CS AAS (Analytik Jena, Germany) was used for MAS measurements. The spectrometer was equipped with a 300-W xenon short-arc lamp as a continuum radiation source for the wavelength range from 185 to 900 nm, a compact high-resolution double echelle monochromator and a charge-coupled device (CCD) array detector with a resolution of about 2 pm per pixel at 200 nm. For pyrolysis and molecule formation after extraction step, the graphite furnace technique (ET) and pyrolytically coated graphite tubes with integrated platform were employed. The operating parameters of the HR-CS ET MAS instrument during F determination after USA DMSPE are summarised in Table 1.

**Table 1:** Optimised experimental conditions for ultrasound-assisted dispersive micro solid phase extraction (USA DMSPE) with TiO_2_ as an adsorbent coupled to HR-CS ET MAS for F determination (parameters for preparation of real samples and reference materials are also presented)

Sample preparation	
Microwave-assisted digestion of solid samples	ca. 500 mg sample, 1 mL 30% H_2_O_2_, 5 mL 65% HNO_3_, 20 min, 300 W
Direct analysis	Water samples
USA DMSPE with TiO_2_	
Sample volume (mL)	10
Amount of TiO_2_ (mg)	20
pH of sample solution	4.0
Ultrasonication time (s)	10
Centrifugation time (min)/rpm	3/4500
Solution for TiO_2_ slurry/final volume (μL)	H_2_O/200
HR-CS ET MAS detection	
Wavelength (nm)	606.440
Lamp current (A)	9
Spectral range (pixel)	200
Dispersion (pm pixel^−1^)	2
Read time (s)	5
Delay time (s)	0
Measurement mode	Peak height
Sample volume (μL)	20
Molecule-forming element/amount (μg)	Ca/24
Furnace program steps	
Drying	80 °C, ramp 6 °C s^−1^, hold 20 s
Drying	90 °C, ramp 3 °C s^−1^, hold 20 s
Drying	120 °C, ramp 5 °C s^−1^, hold 10 s
Pyrolysis	700 °C, ramp 300 °C s^−1^, hold 10 s
Molecule formation	2450 °C, ramp 2500 °C s^−1^, hold 5 s
Cleanout	2550 °C, ramp 500 °C s^−1^, hold 4 s

For SPE procedure, TiO_2_ nanoparticles were weighed using an M2P micro analytical balance (Sartorius, Gottingen, Germany) with a resolution of 1 μg (electronic weighing range up to 2 g). The pH values were measured with a pH meter (pH 211 Microprocessor, Hanna Instruments, Kehl, Germany) supplied with a glass-combined electrode. A Sonopuls HD 70 ultrasonic cell disruptor/homogeniser (70 W, 20 kHz, Bandelin, Germany) equipped with a 2-mm titanium microtip was used for dispersive extraction processes as well as for slurry preparation before MAS detection. Ultrasonic energy was fixed at 40 W.

A UniClever focused microwave sample preparation system (Plazmatronika, Wrocław, Poland) operating at 2450 MHz and 300 W maximum output was used for certified reference materials and real sample digestion before F determination. The high-pressure TFM-PTFE vessel capacity was 110 mL, and the maximum pressure and maximum temperature were 100 atm and 300 °C, respectively.

### Reagents and solutions

Compressed argon of UHP 5.5 purity obtained from Air Products (Warsaw, Poland) was employed as a carrier gas without further purification. Standard solutions of fluoride were prepared from 1000 mg L^−1^ NaF standard solution traceable to SRM from NIST (Certipur, Merck). Ca(NO_3_)_2_·4H_2_O for analysis (EMSURE, Merck) was used as a calcium source and appropriate molecule-forming element. All working standard solutions were prepared daily to prevent any possible changes. The appropriate stock solution was diluted with high-purity water.

Titanium dioxide nanopowder TiO_2_ (≥ 99.5%, P25 Aeroxide, Degussa, Germany) with spherical particles (10–30 nm) was used as an adsorbent in dispersive micro solid phase extraction. Its average particle size was approximately 21 nm and specific surface according to BET 50 ± 15 m^2^ g^−1^ with an anatase/rutile ratio of about 80:20. The pH of the sample solutions was adjusted with 65% HNO_3_ and 30% NaOH (Suprapur, Merck). HNO_3_ (65%) and H_2_O_2_ (30%) (Suprapur, Merck) were used for digestion of the samples. High-purity water: deionised and doubly distilled water (quartz apparatus, Bi18, Heraeus, Germany) were used throughout the experiments.

### Standard reference materials and real samples

The accuracy of the analytical procedure described in this work was verified using certified reference materials (CRMs). The following materials were chosen: INCT-MPH-2 (Mixed Polish Herbs) and INCT-SBF-4 (Soya Bean Flour) from the Institute of Nuclear Chemistry and Technology (Warsaw, Poland); ERM-CAO11b (Hard Drinking Water) supplied by LGC Limited (Teddington, UK); and TMDA-54.5 (Lake Ontario Water) supplied by Environment Canada (Burlington, Canada).

The certificates of the materials used do not contain certified values for the analyte. The materials were analysed before and after spiking with fluoride.

In the course of the study, the analyte was extracted and determined in three herbs samples: burdock (*Arctium lappa*), yarrow (*Achillea millefolium*) and horsetail (*Equisetum*)*.* The samples were collected in the vicinity of Poznań (west-central Poland, on the Warta river), dried thoroughly, ground in an agate mortar and digested before further extraction procedure.

The water samples (lake, tap sample no.1 and well water) were collected in Nysa, a town in southwestern part of Poland on the Nysa Kłodzka River. The lake samples (nos. 1–4) were collected from different sampling points situated on the Nyskie Lake. The place of sampling was chosen not accidentally. The Neogene aquifer has been the main source of drinking water for Nysa and the surrounding area for years. However, due to the high fluoride content, the water supplies abstracting the Neogene aquifer water were closed. High fluoride content in water from the aquifer has caused fluorosis disease in the population of Nysa. Fluoride in water from this region originates from a long-time contact of the water with Precambrian and Palaeozoic igneous rocks composed of fluorine-rich minerals. For comparison, the samples of sea water (Baltic, Adriatic and Tyrrhenian) and tap water (sample no. 2) from the laboratory (Poznań University of Technology) were analysed.

### Analytical procedures

#### Microwave-assisted digestion of solid samples before extraction step

Approximately 500 mg (herbs, soya flour) of powdered sample was placed in the TFM-PTFE vessel of the microwave digestion system and moistened by 1 mL of 30% H_2_O_2_. Then, 5 mL of 65% HNO_3_ was added. The samples were heated for 20 min at 300 W.

Then, the clear digested solution was transferred into 10 mL calibrated flask and diluted to volume with high-purity water. Before further analysis, this solution was appropriately diluted depending on the concentration level of the analyte. In all cases, a corresponding blank was also prepared according to the above microwave-assisted digestion procedure. After this procedure, it was possible to conduct the extraction procedure.

#### Ultrasound-assisted dispersive micro solid phase extraction

Ten millilitres of a sample solution was poured into a centrifuge tube. The pH of the sample was adjusted to 4.0. Then, 20 mg of TiO_2_ (as 200 μL of 10% m/v suspension) was added. The suspension (4 mL containing 400 mg of TiO_2_) was prepared before analysis with the use of ultrasound (2-mm titanium microtip, 40 W, 20 kHz, for 40 s). Subsequently, the sample solution with nanomaterial was ultrasonicated for 10 s. Homogenisation was achieved that promoted the interaction between F and the nanoparticles. After that, the mixture was centrifuged for 3 min at 4500 rpm. The analyte adsorbed on TiO_2_ settled on a bottom of the tube. A water phase was removed and solid phase was mixed with 200 μL of H_2_O and treated with ultrasound for 5 s (ultrasonic probe; 2-mm titanium microtip, 40 W, 20 kHz) to achieve a slurry solution. In order to determine F in the TiO_2_ slurry solution, 20 μL of the solution was injected for HR-CS ET MAS determination under the optimised conditions. Optimised experimental conditions for USA DMSPE with TiO_2_ as an adsorbent coupled to HR-CS ET MAS for fluorine determination are presented in Table 1.

#### Fluorine determination using HR-CS ET MAS

The graphite furnace temperature program used for the determination of F via their diatomic molecules CaF after USA DMSPE is given in Table 1. For analysis, 20 μL of the sample followed by 2 μL of a calcium nitrate (Ca(NO_3_)_2_·4H_2_O) solution at a concentration of 12 g L^−1^ was directly injected onto the platform of the graphite tube. Calibration was performed by the standard calibration technique using standards after ultrasound-assisted dispersive micro solid phase extraction.

## Results and discussion

### Optimisation of HR-CS ET MAS detection

#### Selection of wavelength

The formation of a competitive molecule other than the desirable molecule of the analyte is one of the potential sources of error in MAS. It reduces the concentration of the desirable molecule in the gas phase and affects the sensitivity. Due to the relatively high concentrations of calcium in the water and food samples, we decided to investigate the possibility of using CaF molecular absorption for analytical purposes. According to the literature data this diatomic molecule is stable (its bond dissociation energy is around 529 kJ mol^−1^) and the most intensive band head is found at 606.440 nm, which is part of the X^2^Σ^+^-A^2^Π electronic transition [33, 34]. The molecular absorption band is in the visible range of the spectrum where only very few atoms have absorption lines. It minimises the possible spectral interferences. However, there is the possible interference caused by Cl. A broadband of CaCl formed in the presence of Cl overlapped with the CaF line at 606.440 nm [35]. In our study, no significant interferences were observed. It can be due to the effective separation of the analyte from the matrix during preconcentration step. Additionally, calcium used as the molecule-forming reagent acts also as a chemical modifier, so that no other reagent had to be added.

#### Optimisation of calcium amount

There are many reports in the literature on the use of several elements (Al [17–19], Ga [20–25], Ca [26–28] or Sr [29]) to formation of fluoride diatoms. In general, the reaction with Ga allows to achieve the lowest detection limits. However, this procedure requires the use of additional permanent modifiers of a graphite tube. Coating with zirconium, magnesium, palladium, ruthenium or tungsten is usually used [21–25]. It extends the time of analysis and increases the amount of reagents needed. At the same time, a choice of reagent should be connected with the composition of the sample matrix. Some authors have even investigated the use of Ca originally present in food samples to carry out this reaction [27]. Additionally, the works already published indicate that CaF does not require the use of additional modifiers of a graphite tube as it is with GaF [27]. This is why, for food and water samples, we decided to choose Ca as a molecule-forming element.

**Fig. 1:**
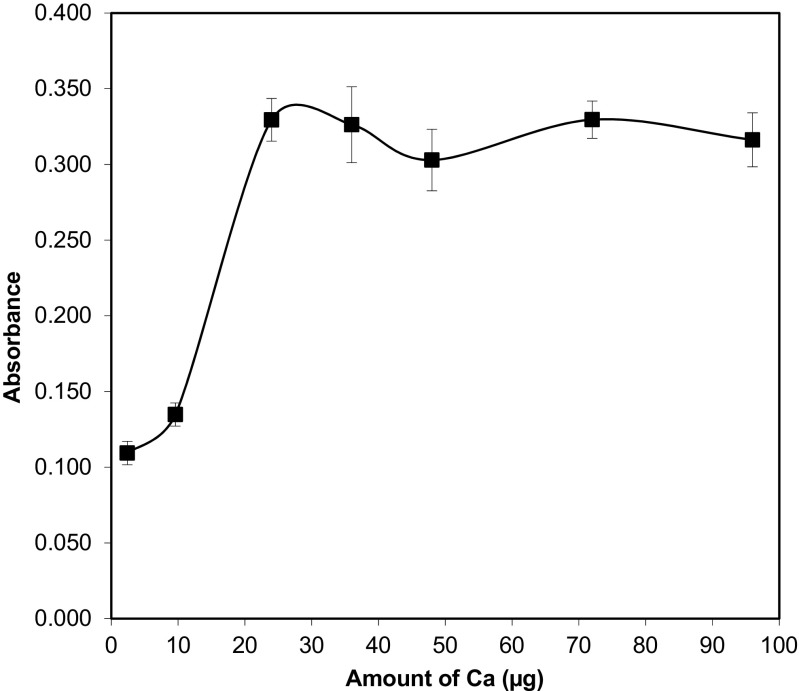
The effect of Ca amount on the CaF molecular absorption. Conditions: standard solution of F^−^ (50 ng mL^−1^) analysed after USA DMSPE, pH = 3.5, sample volume 10 mL, ultrasonication time 10 s, centrifugation 4 min. The error bar is the standard deviation (SD, *n* = 3)

The efficiency of formation of CaF highly depends on the amount of calcium which should be high enough to provide constant and maximum sensitivity for F concentrations in all samples. Therefore, the influence of calcium amount on the analytical signal was investigated within the range of 2.4–96 μg. In this study, 2 μL of a calcium nitrate (Ca(NO_3_)_2_·4H_2_O) solution at a concentration of 12 g L^−1^ was used. The absorbance increased with an amount of calcium in the range of 2.4–24 μg (Fig. 1). For an amount of calcium equal to 24 μg, the absorbance reached its maximum and was found to have a plateau in the range 24–96 μg. Therefore, in this study, the quantity of 24 μg of calcium was chosen for further experiments. The molar ratio between calcium and fluorine ([Ca]:[F]) was found to be about 11 for 20 μL of standard solution at a concentration of 50 ng mL^−1^.

#### Optimisation of pyrolysis and molecule-forming temperature

**Fig. 2:**
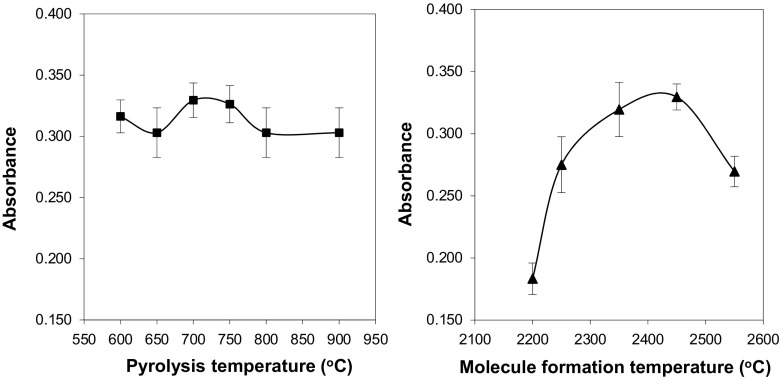
Pyrolysis and molecule formation curves for CaF. Conditions: standard solution of F^−^ (50 ng mL^−1^) analysed after USA DMSPE, for pyrolysis runs Tmf 2400 °C, for molecule formation runs Tp 700 °C. The error bar is the standard deviation (SD, *n* = 3)

The temperature program was optimised for a standard solution containing 50 ng mL^−1^ of fluoride after preconcentration on TiO_2_. Three drying steps were necessary in order to avoid spattering of the sample and to obtain a uniform solid deposit on the graphite surface. The effect of pyrolysis temperature on absorbance was studied within the range 600–900 °C (Fig. 2). No significant differences between analytical signals were observed. However, the absorbance obtained for 700 °C was relatively the highest and this value of the pyrolysis temperature was chosen for further experiments. After optimisation of pyrolysis conditions, the effect of molecule-forming temperature on analytical signals was studied within the range 2200–2650 °C (Fig. 2). Maximum absorbance was achieved at a temperature of 2450 °C. Complete vaporisation and CaF molecule formation were achieved in the graphite tube with minimal influence exerted by the matrix. The temperature program used for F determination in certified reference materials and real samples is shown in Table 1.

Some elements (e.g. vanadium, titanium, molybdenum) form stable carbides in the presence of graphite at high temperatures. Carbide formation can inhibit the decomposition process of the graphite tube and affect the analytical reproducibility and sensitivity or give a significant memory effect. In the course of the study, the pyrolytically coated graphite tube was used. It made the atomizer relatively unreactive chemically and reduced the tendency for carbides formation. Relative standard deviation (RSD) values recorded during the study were found to be satisfactory; therefore, the effect of reaction of C and TiO_2_ was considered to be neglected.

### Optimisation of TiO_2_ USA DMSPE conditions

The parameters that affect the extraction efficiency, including the amount of TiO_2_, the sample pH, homogenisation (ultrasonication) and centrifugation time as well as a slurry preparation step before injection to HR-CS ET MAS were investigated. Initial conditions with the analyte concentration of 50 ng F^−^ mL^−1^ and sample volume of 10 mL were used. Regarding the amount of applied TiO_2_, 4 mL of a suspension containing 400 mg was prepared. Of the suspension added to the sample solution, 200 μL corresponded to 20 mg of TiO_2_ per sample. All the experiments were performed in triplicate (*n* = 3). Due to the construction of the centrifuge and vials, it was not possible to apply higher sample volume.

#### Particle amount

**Fig. 3:**
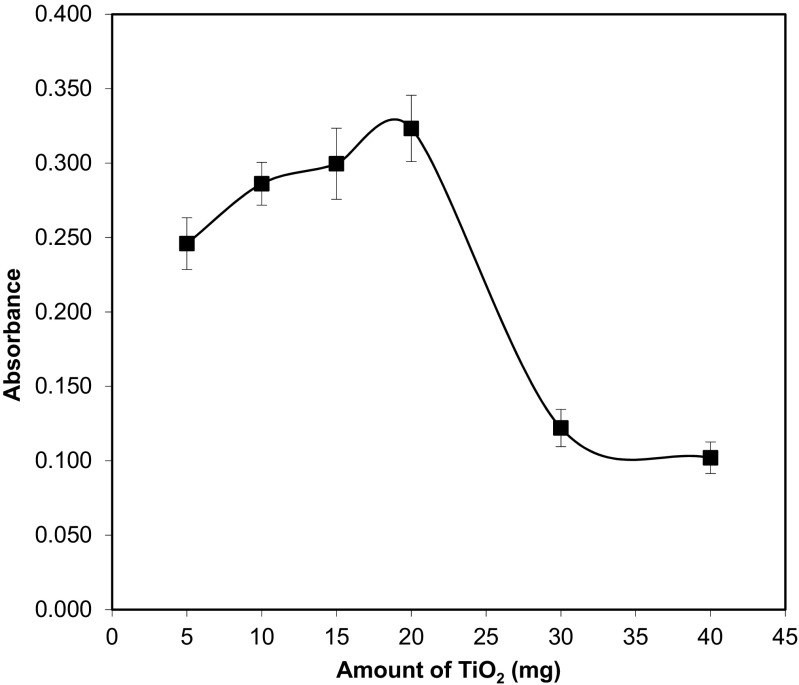
The effect of amount of TiO_2_ on the determination of F with the use of USA DMSPE procedure. Conditions: standard solution of F^−^ (50 ng mL^−1^), pH = 3.5, sample volume 10 mL, ultrasonication time 10 s, centrifugation 4 min. The error bar is the standard deviation (SD, *n* = 3)

To achieve an optimum amount of TiO_2_ necessary for quantitative enrichment of the analyte, the introduced amount of nanoparticles was varied from 5 to 40 mg per sample (10 mL). It was found that effective adsorption of F (the highest analytical signal) was achieved with amounts of nanomaterial from 20 mg (Fig. 3). As observed, a significant decrease in the signal after exceeding the value of 20 mg of adsorbent is difficult to interpret. It can be caused by too high concentration of a suspension injecting to the furnace and therefore interferences in formation of diatomic molecules CaF. For further application, the amount of TiO_2_ equal to 20 mg was used (that corresponding to 200 μL of 10% TiO_2_ slurry solution added to 10 mL of the sample).

TiO_2_ nanoparticles with 80% of anatase were chosen due to the possible mechanism of interaction with the analyte and possibility of effective adsorption connected with high specific surface area. The adsorbent is known for its physical and chemical stability in acidic and alkaline solutions, low cost, nontoxicity and fast rate of adsorption and desorption [36, 37].

Chemical forms of the adsorbent and the analyte, as well as the nature of the interactions, depend strongly on the pH of the sample and are discussed in section “Sample pH”.

#### Sample pH

**Fig. 4:**
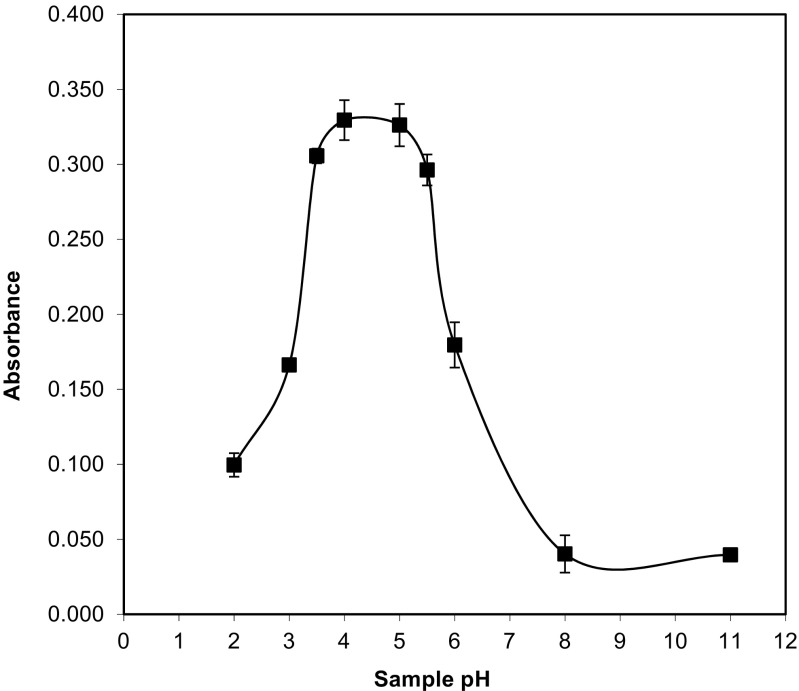
The effect of sample pH on the determination of F with the use of USA DMSPE procedure. Conditions: standard solution of F^−^ (50 ng mL^−1^), sample volume 10 mL, 20 mg of TiO_2_, ultrasonication time 10 s, centrifugation 4 min. The error bar is the standard deviation (SD, *n* = 3)

The pH value of the sample is one of the most important factors affecting the extraction efficiency. The effect of pH on the adsorption of F on nano-TiO_2_ was studied by varying the pH of the aqueous solutions in the range of 2–11. To modify the pH, appropriate amounts of NaOH or HNO_3_ were added to sample solution. The results of the pH optimisation are shown in Fig. 4. It was observed that when the pH was low (1.0–4.0), the adsorption of the analyte (absorbance values) increased. For pH = 4, the absorbance of the analyte reached its maximum value. However, with further increases in the pH (above 5.5), the absorbance decreased significantly for F. Therefore, in this study, a pH of 4.0 was chosen for further experiments.

The adsorption mechanism of the analyte on nano-TiO_2_ depends on the type of the active sites on the adsorbent surface and the concentrations of produced sorbate species at the measured pH value [38]. The point of zero charge (PZC) of the anatase nanoparticles has been found at pH ca. 6.2 [36, 39, 40]. Below this value, the surface of anatase nanoparticles consisted of the mixture of positively charged TiOH_2_^+^ and neutral species of TiOH^0^. The neutral species of TiOH^0^ and the negatively charged TiO^−^ occur in the solution at pH above PZC (basic pH) [41, 42]. It was found that at pH 4.0, the surface of adsorbent is mostly covered with positively charged TiOH_2_^+^ [38].

Fluorine in an aqueous solution has different species, including F^−^, HF and HF_2_^−^. HF is the dominant species at pH 1–3.18, while F^−^ is the dominant species at pH above 3.18. An increase in HF_2_^−^ species is observed as total fluoride concentration increases. However, when the total dissolved fluoride is below 50 mg L^−1^, the maximum fraction of HF_2_^−^ is less than 10% [43, 44]. As a result, there are only two soluble fluoride species considered, HF and F^−^.

According to the theory, the adsorption of anions on titanium dioxide proceeds when the pH of the solution is lower than the point PZC of the oxide (ca. 6.2 of pH) [39, 45]. On the base of literature, the spontaneous adsorption of H^+^ on titania results in a positive charge on the anatase surface and thus the latter becomes active to the adsorption of fluoride [39, 46]. Additionally, pH < 5 is the most favourable for fluoride uptake due to the lower concentration of OH^−^ ions, which compete with fluoride for sorption sites with increasing concentration of OH^−^ in the solution and reduce the capacity for sorption of F^−^. Speciation of the investigated anion is the second reason for lower fluoride sorption at pH *<* 3.18 when it exists as HF. Maximal sorption capacity is observed at pH 4, at which the predominant form of the anion is F^−^. Thus, the adsorption of the analyte can be described as interaction of positively charged TiOH_2_^+^ with F^−^ [44].

#### Ultrasonication and centrifugation time

The positive effect of dispersion on the active area of a solid sorbent used in DMSPE procedure is well known [31, 32, 47]. The dispersion can be chemically achieved (usually using an organic solvent) or assisted by an ultrasound energy. The influence of the external energy source allows for effective reduction of the particles size of the sorbent. This results in a significant increase in the area-to-volume ratio [31, 32, 47].

To investigate the effect of ultrasonication on the extraction process, the dispersion was performed at 40 W ultrasonic power in continuous mode and was evaluated in the range of 5–30 s. An ultrasonic probe (2-mm diameter) was used and the study was conducted for 10 mL of sample solution containing adsorbent and the analyte. During the ultrasonication step, the solutions became milky by dispersing the nanomaterial in the volume of sample. In the first 10 s of extraction, the absorbance increased then after exceeding this time remained approximately constant. Therefore, for further study, an ultrasound-assisted dispersion step of 10 s was chosen.

After the extraction step, the sample was centrifuged to separate the liquid and solid phases.

To investigate the absorbance as a function of centrifugation time, the experiments were performed in the range of 1–6 min (at the rate of 4500 rpm). The volume of sedimented phase (as well as absorbance) increased with increasing time of centrifugation.

A complete settling of the solid phase to the bottom of the centrifuge tube was observed for 3 min. Above this time, the absorbance value was constant. Finally, during the extraction, the sample was centrifuged for 3 min. Thus, 3 min was selected as the final centrifugation time (at the rate of 4500 rpm).

#### TiO_2_ slurry solution preparation

In this research, the injection of slurry samples into the atomizer during HR-CS ET MAS analysis was performed. Therefore, after extraction step, the TiO_2_ slurry containing the analyte was prepared before the analysis. The volume of H_2_O was varied in the range of 150–1000 μL. For a volume of H_2_O, less than 150 μL obtained precision (expressed as RSD) was not satisfactory (above 15%). On the other hand, a decrease of analytical signals was observed for volumes above 250 μL. It was caused by too high dilution of the samples. Therefore, in this study, H_2_O volume of 200 μL was chosen as a solution for slurry preparation before analysis.

For slurry preparation, the water phase was removed after centrifugation. Then, the solid phase was mixed with 200 μL of H_2_O and ultrasonicated for 5 s (2-mm titanium microtip, 40 W, 20 kHz) to achieve a stable slurry solution which was injected to the detector (20 μL) prior to HR-CS ET MAS analysis.

### Analytical figures of merit

The analytical parameters characterising the proposed USA DMSPE HR-CS ET MAS method were investigated under the optimised experimental conditions (Table 1). Seven replicate measurements of the total procedure for standard solution at a concentration of 50 ng mL^−1^ were carried out, and the RSD was calculated. The RSD achieved for F was 7%. The detection limit (LOD) achieved for 10 mL of real sample solution was 0.13 ng mL^−1^. This value was calculated as the concentration of the analyte yielding a signal equivalent to three times the standard deviation of the blank value (*n* = 5). Calibration was performed by the standard calibration technique using standards after USA DMSPE extraction. Received correlation coefficient (0.9961) was acceptable. The preconcentration factor achieved for F was 7. This factor was calculated using the ratio of the analyte concentration in the slurry solution containing solid phase with the analyte to the initial concentration of the analyte within the sample solution [32, 48].

### Accuracy verification

The accuracy of the analytical procedure described in this work was verified using CRMs. The following materials were chosen: INCT-MPH-2 (Mixed Polish Herbs), INCT-SBF-4 (Soya Bean Flour), ERM-CAO11b (Hard Drinking Water) and TMDA-54.5 (Lake Ontario Water). The certificates of the materials used do not contain certified values for the analyte. The materials were analysed before and after spiking with fluoride (4.0 mg kg^−1^, calculated on the mass of solid material and 0.150 or 0.100 mg L^−1^ for water matrixes). After spiking, the solid CRMs were digested before further extraction procedure. The waters were spiking directly before extraction step. The recoveries were between 97 and 109% that is considered satisfactory for the low concentration levels of the analyte (Table 2).

**Table 2:** Determination of F in certified reference materials (CRMs) (before and after spiking) by USA DMSPE coupled to HR-CS ET MAS using the optimised parameters

CRM	Determined				Added	Value of *t* test	Significance^d^
	Total F concentration (mg kg^−1^)	Added	Recovery (%)	RSD (%)	F concentration (mg kg^−1^)		
INCT-MPH-2^a^ Mixed Polish Herbs	13.0 ± 1.4	–	–	11	–	–	–
INCT-MPH-2^b^ Mixed Polish Herbs	17.1 ± 1.2	4.1 ± 0.3	105	7	4.0	0.577	NS
INCT-SBF-4^a^ Soya Bean Flour	10.2 ± 0.8	–	–	8	–	–	–
INCT-SBF-4^b^ Soya Bean Flour	14.6 ± 0.9	4.4 ± 0.3	109	6	4.0	2.309	NS
ERM-CAO11b^a^ Hard Drinking Water	0.809^c^ ± 0.097	–	–	12	–	–	–
ERM-CAO11b^b^ Hard Drinking Water	0.954^c^ ± 0.057	0.145^c^ ± 0.009	97	6	0.150^c^	0.962	NS
TMDA-54.5^a^ Lake Ontario Water	0.269^c^ ± 0.030	–	–	11	–	–	–
TMDA-54.5^b^ Lake Ontario Water	0.375^c^ ± 0.019	0.106^c^ ± 0.005	107	5	0.100^c^	2.078	NS

Recovery and relative standard deviation (RSD) values also are shown. Obtained values: average value ± standard deviation (*n* = 3)

*NS* not significant

^a^Analysed before spiking

^b^Analysed after spiking

^c^Milligrams per litre

^d^Significance of *t* test (*n* = 3) at 95% confidence level, *t*_critical_ = 4.303

The certified reference materials (hard and lake waters) used in the research are characterised by a matrix containing relatively high concentrations (μg L^−1^) of the elements (e.g. Al, Ba, Cr, Cu, Fe, Pb, Sr, Zn), sometimes in the range of milligrams per litre (Ca, K, Mg, Na). The solid materials (herbs and soya matrices) contain also high values of Al, Ca, Cl, Cu, K, Mg, Mn, Na, Ni, Pb, Sr and Zn in the range of milligrams per kilograms or even percent. The obtained recoveries of the analyte, in the presence of these elements, proved that the interferences from foreign ions can be ignored. Due to the satisfactory results, the additional study in terms of matrix influence was not carried out.

Obtained results show that the proposed method can be applied for the preconcentration and determination of F in the samples with complex matrices. The precision expressed as RSD (%) was in the range of 5–7% for the certified reference materials after spiking.

### Fluorine determination in real samples

In order to evaluate the usefulness of the proposed method for determination of F, contents in four water samples (lake, tap, well and sea waters) as well as in herb samples were analysed using previously optimised experimental conditions. First, the water samples were collected in 500-mL Schott Duran glass bottles. Then, they were acidified with HNO_3_ (0.5 mol L^−1^) and stored in the dark at 4 °C. A Cameo syringe filter with a polytetrafluoroethylene membrane and a pore size of about 0.22 mm (GE Water & Process Technologies, USA) was used for sample filtration. Before further extraction procedure, herb samples were digested. Digestion procedure was described in section “Microwave-assisted digestion of solid samples before extraction step”. In section “Analytical procedures”, the analytical procedure used for fluorine preconcentration and determination was described. The RSDs (%) achieved for real samples were 3–15% (Table 3).

**Table 3:** Determination of fluorine in real samples by USA DMSPE coupled to HR-CS ET MAS using the optimised parameters

Sample^a^	Fluorine concentration (mg L^−1^)
Lake water no. 1	0.118 ± 0.007
Lake water no. 2	0.110 ± 0.008
Lake water no. 3	0.132 ± 0.004
Lake water no. 4	0.122 ± 0.006
Tap water no. 1	0.156 ± 0.012
Well water	0.239 ± 0.017
Tap water no. 2	0.173 ± 0.019
Sea water (Baltic)	0.153 ± 0.023
Sea water (Adriatic)	0.319 ± 0.016
Sea water (Tyrrhenian)	0.146 ± 0.007
Burdock	4.0^b^ ± 0.5
Yarrow	8.2^b^ ± 0.5
Horsetail	3.4^b^ ± 0.4

Obtained values: average value ± standard deviation (*n* = 3)

^a^Description of the samples in section “Standard reference materials and real samples”

^b^Milligrams per kilogram

As it was already mentioned, drinking water and food are the primary sources of fluoride intake but exposure mainly depends on the geographical origin of a product [2, 3]. According to the WHO limit, fluoride from water may be harmful if it exceeds 1.5 mg L^−1^ [1, 3, 4].

Gomez et al. [17] found the concentration of fluoride in drinking waters in the range 0.05–1.10 mg L^−1^ and in sea waters 1.30–2.50 mg L^−1^. Ozbek et al. [29] determined the fluoride concentration in bottled drinking waters in the range 0.12–0.25 and in the tap waters 0.97–0.04 mg L^−1^. In the course of the study, the established average concentrations of fluoride in analysed waters were 0.121 and 0.206 mg L^−1^, for lake and seas, respectively. Tap waters contained 0.156 and 0.173 mg L^−1^ and it was below the level announced by WHO (1.5 mg L^−1^) (Table 3). Samples collected from areas (Nysa) where soil contains the high fluoride content (lake water nos. 1–4, tap water no.1) were not characterised by higher concentrations of the analyte.

The levels of F in food items depend mainly on the fluoride contents in the soil and water used for irrigation and on the place of origin [2]. Vegetables and fruits contribute little to exposure as they contain low concentrations of fluoride (e.g. 0.1–0.4 mg kg^−1^). Cow’s milk typically contains also low levels of fluoride, e.g. 0.02–0.05 mg L^−1^. However, tea leaves may contain high levels of F (event up to 400 mg kg^−1^ dry weight) [1]. Unfortunately, there is a lack of scientific reports on the concentration of F in herbs intended for the preparation of infusions. In herb samples analysed by USA DMSPE coupled to HR-CS ET MAS, detected average concentration of fluorine was 5.2 mg kg^−1^ (Table 3).

Fluorine is less determined in food samples because of its relatively low concentrations and thus the requirement for low limits of detection. Therefore, it is difficult to compare the results (for samples as for CRMs before spiking) with literature. Additionally, there is no information about EU legislation setting the maximum concentrations of this element in food products.

### Comparison with other methods

In analytical chemistry, for determination of fluoride, solid phase extraction (SPE) methods have been proposed with different adsorbents. For this purpose, SPE columns with charcoal [8], nanometre-size zirconia (ZrO_2_) [49] and mini-column with polymer-zirconium complex (Zr(IV) with a hydrophilic with an iminodiacetic acid group) [50] were investigated. As an effective adsorbent, magnetic iron oxide nanoparticles (MIONs) [51] were used as well as headspace solid phase microextraction (HS-SPME) with carboxen-polydimethylsiloxane fibre (CAR/PDMS)) [52, 53].

The LOD achieved for F (0.13 ng mL^−1^) is comparable with the LOD obtained using H_2_SO_4_-activated nanometre-size zirconia as a solid sorbent prior to determination of fluoride by ion chromatography [49]. The LOD obtained for F is also superior by about two orders of magnitude to the LODs achieved using magnetic iron oxide nanoparticles prior to spectrophotometric determination of the analyte [51] and HS-SPME with carboxen-polydimethylsiloxane fibre prior to determination by gas chromatography-tandem mass spectrometry [53]. The LOD reported by Wejnerowska et al. [52] is about 50 times worse than that achieved for F when using nano-TiO_2_ as a solid sorbent. The LOD obtained using SPE columns with charcoal for fluoride preconcentration prior to reversed-phase high-performance liquid chromatography (RP HPLC) determination is superior by a factor of 7 to the LOD presented in this study [8].

Additionally, the obtained detection limit was compared with those achieved without the enrichment step, only using MAS detection (Table 4). Unquestionably, the preconcentration procedure conducted by the USA DMSPE allows a significant improvement in the detection limit using MAS technique.

**Table 4:** Comparison of the proposed method with other reported in the literature

Detection technique	Form of fluoride	LOD (μg L^−1^)	Ref.
HR-CS F MAS	AlF	5500	16
ETAAS	AlF	20	17
Solid sampling ET MAS	AlF	0.17^a^	19
HR-CS ET MAS	SrF	36	29
HR-CS F MAS	GaF	1000	20
HR-CS ET MAS	GaF	0.9	21
HR-CS ET MAS	GaF	0.26	22
HR-CS ET MAS	GaF	0.26	23
HPLC-HR-CS ET MAS	GaF	4^b^	24
HR-CS ET MAS	GaF	0.58	25
HR-CS ET MAS	CaF	160	26
HR-CS ET MAS	CaF	26	27
Slurry sampling ET MAS	CaF	2.2^a^	28
HR-CS ET MAS	CaF	380	33
Solid sampling ET MAS	CaF	0.36^a^	34
Slurry sampling ET MAS	CaF	5^a^	35
USA DMSPE HR-CS ET MAS	CaF	0.13	This work

^a^Micrograms per gram

^b^Picogram

## Conclusions

The combination of ultrasound-assisted dispersive micro solid phase extraction, with the use of TiO_2_ as an adsorbent and high-resolution molecular absorption spectrometry with electrothermal vaporisation of the calcium monofluoride for preconcentration and determination of F, was presented. The use of nano-TiO_2_ as a solid sorbent enabled to achieve low detection limit and relatively good preconcentration factor. USA DMSPE is effective and takes place in a relatively short time of about 4 min. The use of ultrasonication to make the solid phase well dispersed allowed to reduce the time of the whole procedure. The extraction procedure does not require the use of organic solvents and detection step can be performed with the use of calibration against aqueous standards (after USA DMSPE). Additionally, the procedure enables to extend the analytical capabilities of the HR-CS ET MAS technique. This is the first published combination of the enrichment step using USA DMSPE with fluorine determination by ET MAS. The procedure makes it possible to achieve lower limits of determination compared to other methods based only on ET MAS detection. Proposed methodology makes ET MAS more competitive compared to other published techniques for fluorine determination. Thus, it can be an alternative to them. The method was successfully applied to F determination in natural waters and herb samples without any significant matrix influence.
